# The efficacy of buprenorphine compared with dexmedetomidine in spinal anesthesia: a systematic review and meta-analysis

**DOI:** 10.1016/j.bjane.2024.844557

**Published:** 2024-09-08

**Authors:** Joao Marcos Cansian, Angelo Zanin D'Angelo Giampaoli, Liege Caroline Immich, André Prato Schmidt, Andrei Sanson Dias

**Affiliations:** a Universidade Federal de Ciências da Saúde de Porto Alegre (UFCSPA), Programa de Residência Médica em Anestesiologia, Porto Alegre, RS, Brazil; b Santa Casa de Porto Alegre, Serviço de Anestesia, Porto Alegre, RS, Brazil; c Hospital de Clínicas de Porto Alegre (HCPA), Serviço de Anestesia e Medicina Perioperatória, Porto Alegre, RS, Brazil; d Hospital Nossa Senhora da Conceição (HNSC), Serviço de Anestesia, Porto Alegre, RS, Brazil; e Universidade Federal do Rio Grande do Sul (UFRGS), Programa de Pós-Graduação em Ciências Pneumológicas, Programa de Pós-Graduação em Ciências Cirúrgicas, Porto Alegre, RS, Brazil; f Universidade de São Paulo (USP), Faculdade de Medicina (FM), Programa de Pós-Graduação em Anestesiologia, Ciências Cirúrgicas e Medicina Perioperatória, São Paulo, SP, Brazil

**Keywords:** Anesthesia, spinal, Buprenorphine, Dexmedetomidine, Pain, Postoperative nausea and vomiting

## Abstract

**Background:**

This study compares dexmedetomidine and buprenorphine as potential adjuvants for spinal anesthesia. Dexmedetomidine enhances sensory block and minimizes the need for pain medication, while buprenorphine, a long-acting opioid, exhibits a favorable safety profile compared to traditional opioids.

**Methods:**

PubMed, Cochrane and EMBASE were systematically searched in December 2023. Eligibility criteria: RCTs with patients scheduled for lower abdominal, pelvic, or lower limb surgeries; undergoing spinal anesthesia with a local anesthetic and buprenorphine or dexmedetomidine.

**Results:**

Eight RCTs involving 604 patients were included. Compared with dexmedetomidine, buprenorphine significantly reduced time for sensory regression to S1 (Risk Ratio [RR = -131.28]; 95% CI -187.47 to -75.08; I^2^ = 99%) and motor block duration (RR = -118.58; 95% CI -170.08 to -67.09; I^2^ = 99%). Moreover, buprenorphine increased the onset time of sensory block (RR = 0.42; 95% CI 0.03 to 0.81; I^2^ = 93%) and increased the incidence of postoperative nausea and vomiting (RR = 4.06; 95% CI 1.80 to 9.18; I^²^ = 0%). No significant differences were observed in the duration of analgesia, onset time of motor block, time to achieve the highest sensory level, shivering, hypotension, or bradycardia.

**Conclusions:**

The intrathecal administration of buprenorphine, when compared to dexmedetomidine, is linked to reduction in the duration of both sensory and motor blocks following spinal anesthesia. Conversely, buprenorphine was associated with an increased risk of postoperative nausea and vomiting and a longer onset time of sensory block. Further high-quality RCTs are essential for a comprehensive understanding of buprenorphine's effects compared with dexmedetomidine in spinal anesthesia.

## Introduction

Local anesthetics stand out for their effectiveness in blocking sensory and motor stimuli. However, their use is not without risks, as it can be associated with systemic toxicity, manifesting through a broad spectrum of signals and symptoms, ranging from neurological to cardiac manifestations, ultimately leading to cardiac arrest and, in severe cases, death.^1^ Additionally, when local anesthetics are used in regional anesthesia, they often induce some degree of sympathetic blockade.^2^ With a focus on maximizing clinical benefits, researchers are actively exploring adjuvants for local anesthetics in spinal anesthesia. These innovations aim to enhance efficacy, alleviate side effects, and ensure exceptional perioperative analgesia.^3^

Morphine was the inaugural opioid employed for intrathecal anesthesia in the early 20^th^ century.^4^ It continues to find extensive use as a local anesthetic adjuvant, sharing similar side effects with fentanyl and sufentanil. However, morphine differs in that it has an increased likelihood of inducing ventilatory depression and cephalic spreading due to its increased hydrophilicity (fentanyl and sufentanil are more lipophilic).^1^ Theoretically, opting for intrathecal administration of opioids over intravenous delivery offers several advantages, such as the potential for using smaller doses. This approach may effectively diminish pain sensation without inducing autonomic changes or compromising motor function and sensation. Additionally, the specific opioid antagonist naloxone can be administered to counteract any undesired effects.

Buprenorphine, an agonist-antagonist opioid, stands out for having the longest duration among opioids used in clinical settings, with a half-life ranging from 2 to 16 hours after intravenous administration and 24 to 69 hours after sublingual intake. It is capable of producing effects similar to other opioids, including analgesia, sedation, euphoria, and respiratory depression, albeit to a lesser extent than morphine. This characteristic increases the safety margin compared to classical opioids.^2^ Furthermore, buprenorphine exhibits local anesthetic properties, capable of blocking voltage-gated sodium channels. While this effect has been observed with other opioids used in neuraxial anesthesia, it is more pronounced with buprenorphine.^5^ Some studies have noted systemic absorption and an antihyperalgesic effect, highlighting these as positive characteristics.^6^ Intrathecal use of buprenorphine has confirmed significantly longer analgesic effects, albeit with a higher frequency of nausea and vomiting.^7^

Dexmedetomidine, an α2 agonist, can be used as an adjuvant in neuraxial anesthesia. When combined with local anesthetics at clinical dosages ranging from 5 to 10 μg, it has been linked to extended duration of sensory block, improved postoperative analgesia, reduced requirement for rescue analgesics, and prolonged motor block duration. However, it is essential to note that this combination may increase the incidence of reversible bradycardia.^7^

While there are existing meta-analyses focused on studying the effect of dexmedetomidine as an adjuvant in spinal anesthesia, there is no systematic review directly comparing the effects of buprenorphine as an adjuvant in spinal anesthesia with dexmedetomidine. Therefore, this systematic review aimed to compare the efficacy of buprenorphine and dexmedetomidine as adjuvants to local anesthetics in spinal anesthesia. It is important to note that both buprenorphine and dexmedetomidine have not received official approval for neuraxial use from public agencies such as the FDA (Food and Drug Administration) and are therefore used off-label.^8^

## Methods

The study protocol was registered and published on January 7^th^, 2024, on the International Prospective Register of Systematic Reviews (PROSPERO) of the National Institute for Health Research (NIHR) under ID CRD42024498382. We conducted this systematic review and meta-analysis following the Preferred Reporting Items for Systematic Review and Meta-Analysis Protocols (PRISMA) statement recommended checklist.^9^ The comprehensive review of literature and RCTs was conducted by the authors between December 2023 and January 2024.

### Eligibility criteria

Inclusion in this systematic review was restricted to studies that met the following criteria: (a) Patients scheduled for lower abdominal, pelvic, or lower limb surgeries; (b) Patients undergoing spinal anesthesia with a local anesthetic and one of two adjuvants, buprenorphine or dexmedetomidine; (c) Only Randomized Controlled Trials (RCTs) were considered; (d) Studies that reported any comparable outcome of interest. Studies were excluded if there was no comparison group of interest or if patients were aged under 18 years.

### Search strategy and data extraction

We systematically searched for articles meeting the inclusion criteria on MEDLINE, EMBASE, and Cochrane databases. The searches were conducted in December 2023 by two independent investigators. In case of any disagreement regarding the included articles, a third investigator, with better expertise, was consulted to make the final decision. The search strategy was designed to be comprehensive enough to encompass all randomized controlled trials conducted under the aforementioned inclusion criteria. Grey literature and references from all included studies, as well as previous systematic reviews and meta-analyses, were also manually searched.

The search strategy comprised two steps: an electronic search using the terms ((buprenorphine AND dexmedetomidine AND [spinal OR intrathecal]) and a manual search of the reference lists of all studies identified. This manual search process continued until no new articles meeting our inclusion criteria were found.

### Endpoints

All endpoints related to time were measured in minutes. The primary outcome was duration of sensory block, defined as the time of regression to S1 from the maximum sensory block level. Secondary endpoints included: onset time of motor block; onset time of sensory block; duration of motor block; duration of analgesia; and time to peak sensory level. Moreover, the following adverse effects during the procedure or immediately after its execution were recorded: Postoperative Nausea and Vomiting (PONV); bradycardia; hypotension; and shivering.

The Modified Bromage Scale was used by studies for standardization in order to evaluate the grade of motor block, defined as follows: Bromage 0 – the patient is able to move the hip, knee, and ankle; Bromage 1 – not able to move the hip but able to move the knee and ankle; Bromage 2 – not able to move the hip and knee but able to move the ankle; Bromage 3 – not able to move the hip, knee, and ankle.

Authors considered the onset time of motor block as the time taken from the injection of the drug into the intrathecal space until modified Bromage 3; the onset time of sensory block was considered as the time between intrathecal injection to the T12 or higher dermatome; the duration of motor block was defined as the time of regression to modified Bromage score of 0; the duration of analgesia or pain-free period was defined as the time from intrathecal injection to the first time of complaint about pain or rescue analgesia; the time to peak sensory level was defined as the time to the highest dermatomal level of sensory block (sensory level in the RCTs was measured by the pinprick method).

### Subgroup analyses

Variation in buprenorphine, dexmedetomidine and local anesthetics dosing existed among some studies, and this variability was explored in a subgroup analysis when appropriate. To identify potential causes for the elevated heterogeneity in certain outcomes, a subgroup analysis was conducted when heterogeneity fell within the range of 50% to 70% (classified as “substantial heterogeneity”) or greater.^10^

### Quality assessment and risk of bias

The quality assessment of RCTs was conducted using the Cochrane Collaboration's tool for assessing the risk of bias in randomized trials (RoB2). Studies were categorized as having a high, low, or moderate risk of bias in five domains: selection, performance, detection, attrition, and reporting biases. Publication bias was examined through funnel-plot analysis, and estimates were determined based on study weights.

### Statistical analysis

The statistical analysis for this systematic review and meta-analysis adhered to the guidelines set by the Cochrane Collaboration and the Preferred Reporting Items for Systematic Reviews and Meta-Analysis (PRISMA) statement.^9^ Risk Ratio (RR) with 95% Confidence Intervals was employed to compare treatment effects for both categorical and continuous endpoints. A random-effect model was used for outcomes. Heterogeneity was assessed using the Cochrane Q test and I^2^ statistics, where *p*-values less than 0.10 and I^2^ greater than 50% were considered indicative of significant heterogeneity. Review Manager 5.4 (Cochrane Centre, The Cochrane Collaboration, Denmark) was the tool used for statistical analysis.

## Results

### Study selection and characteristics

**Figure 1:**
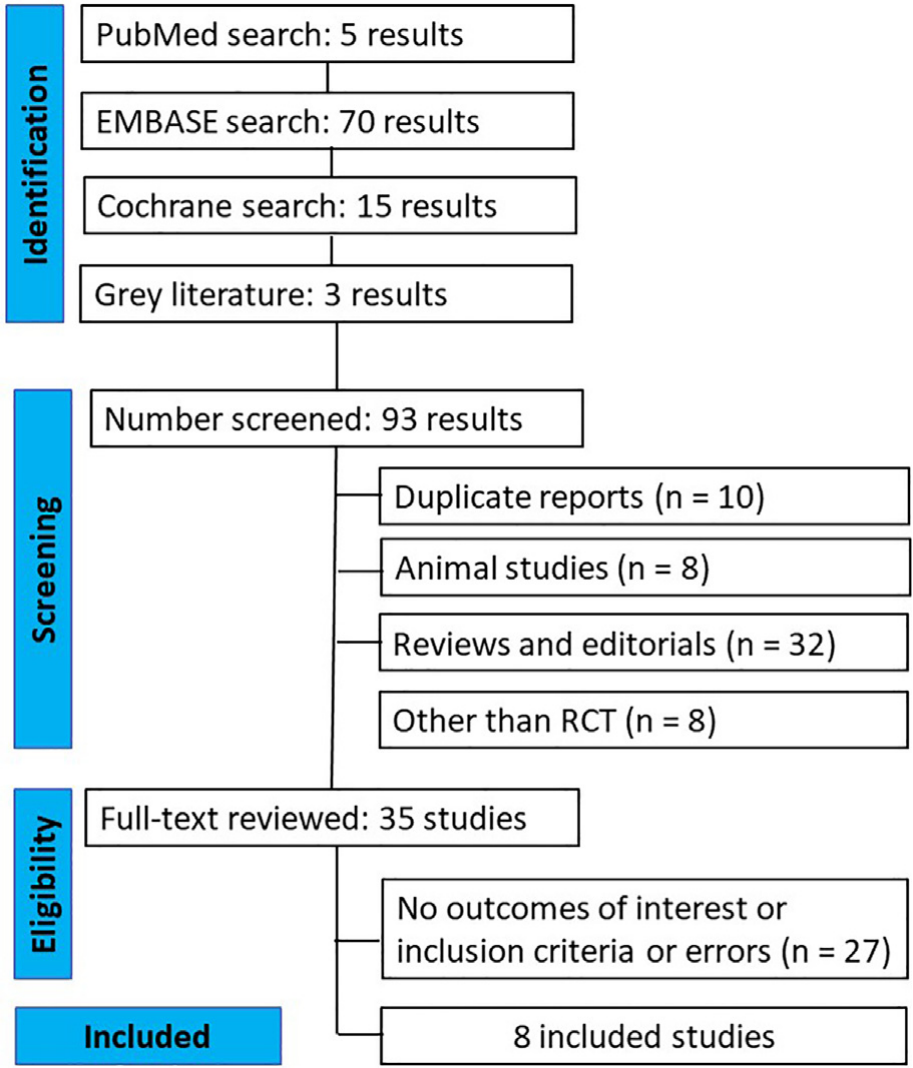
Flow chart of the study selection process. RCT, Randomized Controlled Trial.

The initial search yielded 93 results, as illustrated in Figure 1. Following the removal of duplicate records and ineligible studies, 83 records remained, all of which were thoroughly reviewed against the inclusion criteria. After analysis, a total of 8 RCTs were included, encompassing 604 patients (Table 1).

**Table 1:** Characteristics of included studies.

Study	Year	Patients BUP/DEX	ASA	Study design	Buprenorphine dosing [μg]	Dexmedetomidine dosing [μg]	Local anesthetic / dosing	Type of surgery
Gupta	2014	30/30	I, II	RCT	60	5	HB / 9 mg	Lower abdomen
Kaur	2017	20/20	I, II, III	RCT	60	5	HB / 15 mg	TURP
Akhila	2017	34/34	I, II	RCT	75	5	HB / 12.5 mg	Infra-umbilical
Amitha	2017	30/30	I, II	RCT	30	5	HB / 15 mg	Lower limbs
Deepa	2018	30/30	I, II	RCT	75	5	LB / 15 mg	Lower abdomen / Lower limbs
Gitte	2022	50/50	I, II	RCT	75	10	HB / 15 mg	NS
Ishan	2022	75/75	I, II, III	RCT	75	5	HB / 20 mg	Lower limbs
Gowrilakshmi	2023	35/35	I, II	RCT	30	15	RV 22.5 mg	Infra-umbilical

ASA, American Society of Anesthesiologists physical status; BUP, Buprenorphine; DEX, Dexmedetomidine; HB, Hyperbaric Bupivacaine; LB, Levobupivacaine; RV, Ropivacaine; RCT, Randomized Controlled Trial; TURP, Transurethral Resection of the Prostate.

### Pooled analysis of all studies

**Figure 2:**
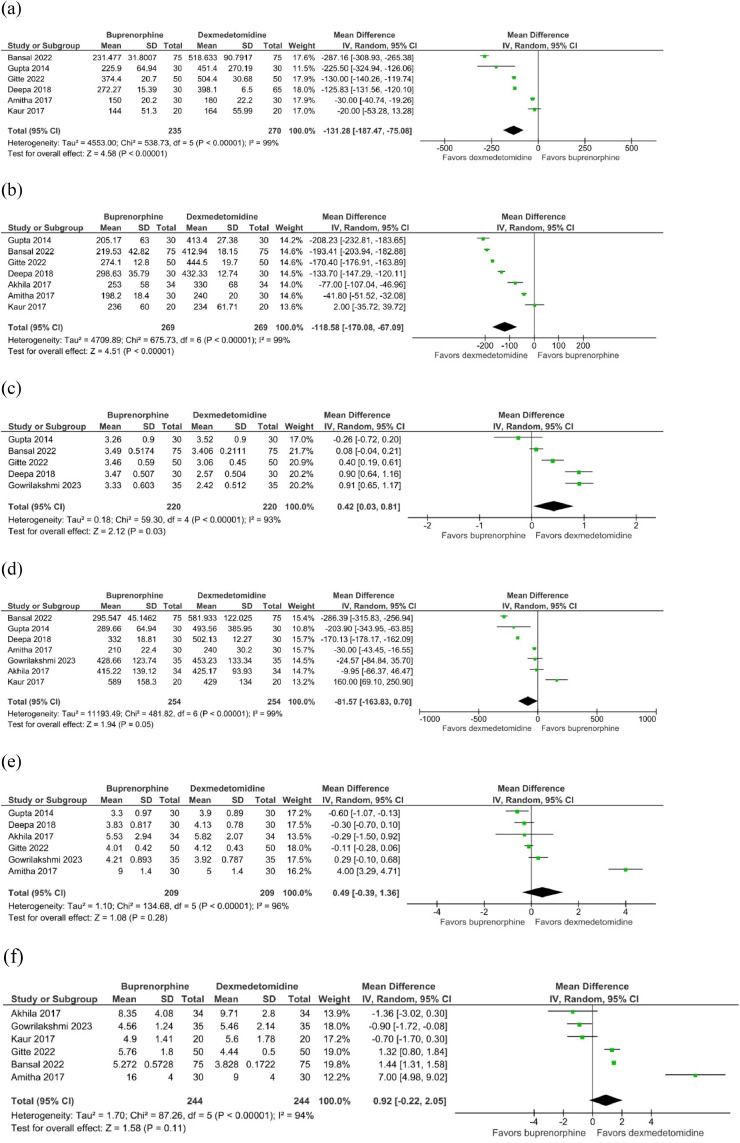
Comparison of time-related variables between the buprenorphine and dexmedetomidine groups: (a) Time of sensory regression to S1; (b) Duration of motor block; (c) Onset time of sensory block; (d) Duration of analgesia; (e) Onset time of motor block; (f) Time to achieve the highest sensory level.

In comparison to dexmedetomidine, buprenorphine was associated with a significant reduction in both the time of sensory regression to S1 (RR = -131.28, 95% CI -187.47 to -75.08; *p* < 0.00001; I^2^ = 99%; Figure 2 a) and the duration of motor block (RR = -118.58, 95% CI -170.08 to -67.09; *p* < 0.00001; I^2^ = 99%;Figure 2 b). Conversely, the onset time of sensory block (Figure 2 c) was slightly delayed, yet still statistically significant (RR = 0.42, 95% CI 0.03 to 0.81; *p* = 0.03; I^2^ = 93%). Notably, no significant differences were observed in the duration of analgesia (RR = -81.57, 95% CI -163.83 to 0.70; *p* = 0.05; I^2^ = 99%; Figure 2 d), onset time of motor block (RR = 0.49, 95% CI -0.39 to 1.36; *p* = 0.28; I^2^ = 96%; Figure 2 e), or time to achieve the highest sensory level (RR = 0.92, 95% CI -0.22 to 2.05; *p* = 0.11; I^2^ = 94%; Figure 2 f).

**Figure 3:**
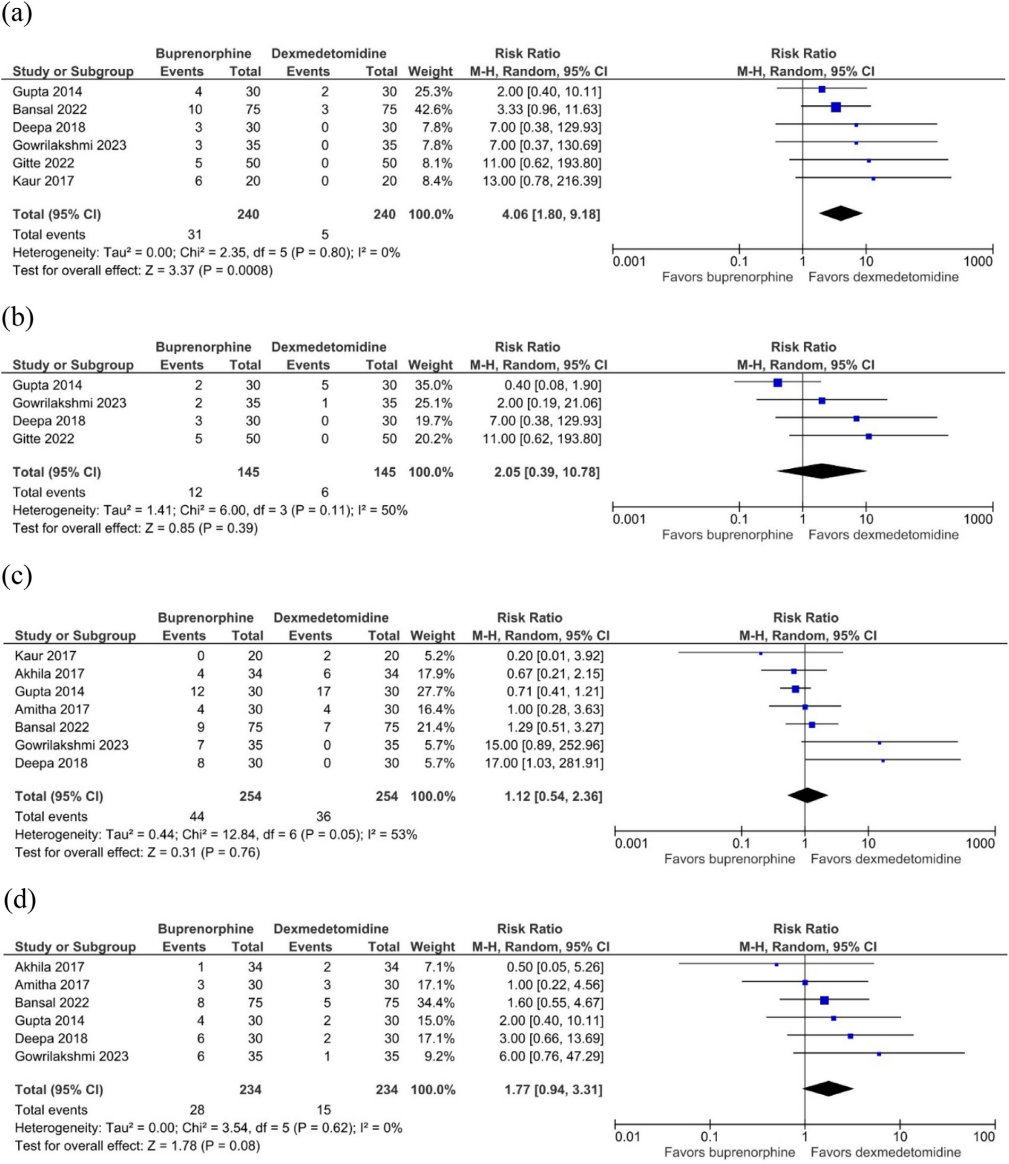
Comparison of the incidence of adverse effects between the buprenorphine and dexmedetomidine groups: (a) Postoperative nausea or vomiting; (b) Shivering; (c) Hypotension; (d) Bradycardia.

When examining adverse effects, no significant differences between groups were found regarding the risk of developing shivering (RR = 2.05, 95% CI 0.39 to 10.78, *p* = 0.39, I^2^ = 50%; Figure 3 b), hypotension (RR = 1.12, 95% CI 0.54 to 2.36, *p* = 0.76, I^2^ = 53%; Figure 3 c), and bradycardia (RR = 1.77, 95% CI 0.94 to 3.31, *p* = 0.08, I^2^ = 0%; Figure 3 d). However, a significant increase in the risk of developing PONV was observed in the buprenorphine group (RR = 4.06, 95% CI 1.80 to 9.18, *p* = 0.0008, I^2^ = 0%; Figure 3 a).

### Subgroup analyses and heterogeneity

Endpoints such as PONV and bradycardia exhibited an I^2^ of zero, rendering subsequent subgroup analysis infeasible. Moderate heterogeneity in shivering analysis precluded subgroup exploration. Moreover, subgroup analysis of hypotension failed to establish statistically significant differences among buprenorphine, dexmedetomidine, or local anesthetic subgroups. Thus, categorical variables were not analyzed by subgroups.

**Figure 4:**
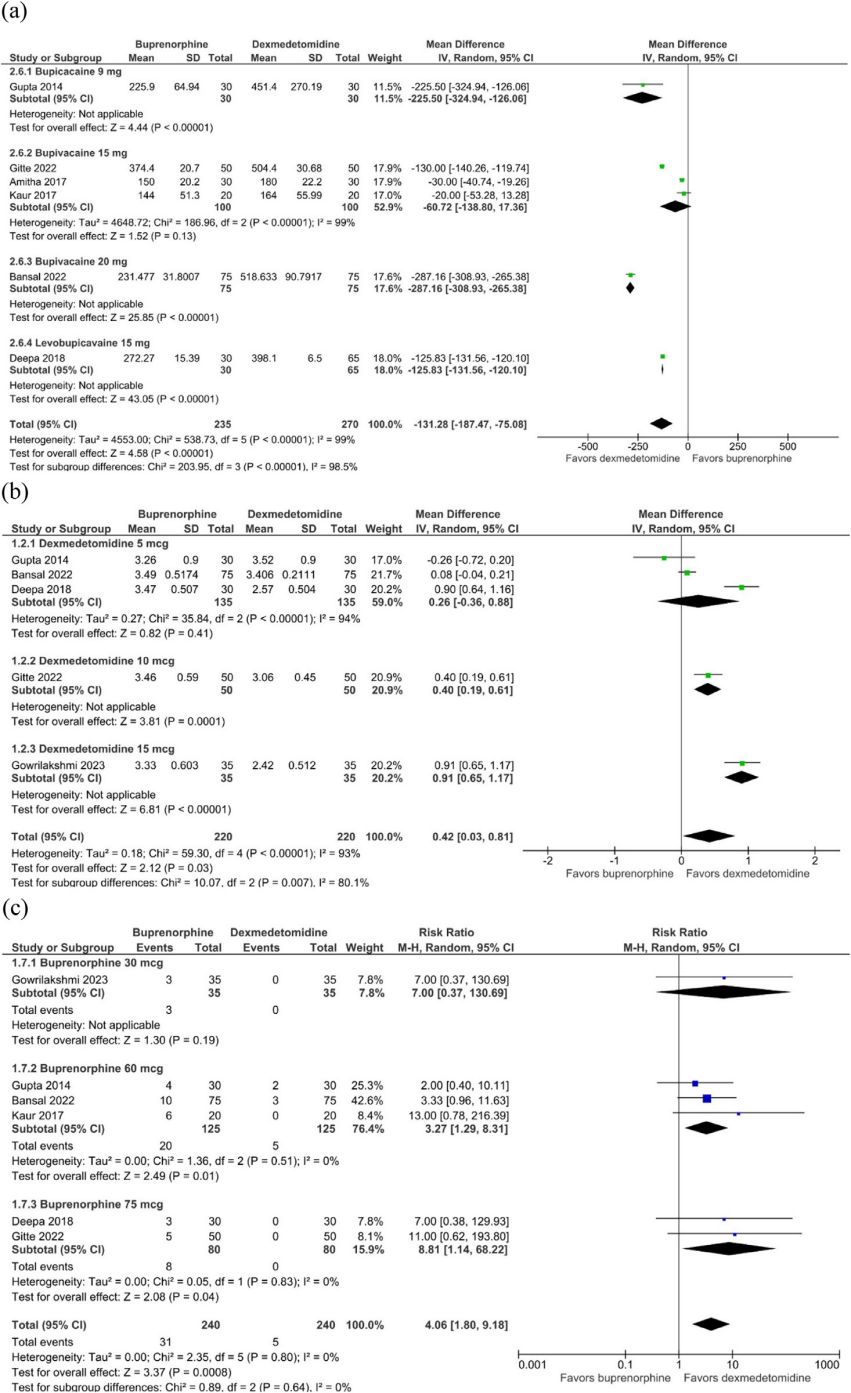
Subgroup analysis: (a) Comparison of different doses of local anesthetics in the time of sensory regression to S1; (b) Comparison of different doses of dexmedetomidine in the onset time of sensory block; (c) Comparison of the dosing effect of buprenorphine in the incidence of postoperative nausea and vomiting.

Subgroup analysis of time-related outcomes was also evaluated. In the primary endpoint, sensory regression to S1, subgroups of local anesthetics dosing and type were statistically different (Figure 4 a; test for subgroup differences with *p* < 0.00001, I^2^ = 98.5%). However, it was not possible to identify a clear interaction between doses of local anesthetics and main outcomes. Conversely, a discernible trend suggested that increasing doses of dexmedetomidine might correlate with a decrease in the onset time of sensory block (Figure 4 b; test for subgroup differences with *p* < 0.007, I^2^ = 80.1%). Additionally, subgroup analysis of different buprenorphine doses and the incidence of PONV did not demonstrate any difference among groups (Figure 4 c; test for subgroup differences with *p* = 0.64, I^2^ = 0%). The remaining subgroup analyses performed over time-related outcomes could not identify any reasonable relationship or explanation for the observed elevated heterogeneity. Nonetheless, differences in drug doses between study arms emerged as a potential contributor to the high heterogeneity in some endpoints.

The limited number of studies included in the meta-analysis may have also played a role in these results. As this meta-analysis features less than ten studies in the screening step, exploration of meta-regression to examine heterogeneity in certain endpoints was not feasible, in line with the existing literature. Additionally, the relatively low number of RCTs precluded the performance of an Egger's Test to examine funnel plot asymmetry.^10^

### Quality assessment and publication bias

Individual RCT appraisal is detailed in Table 2. Of the included studies, four were deemed to have a low risk of bias by RoB2.^11^, ^12^, ^13^, ^14^ Conversely, three studies were classified as high risk of bias, with Deepa et al^3^ losing a point concerning potential bias in the randomization process. The RCTs of Deepa et al,^3^ Gitte et al,^15^ and Gowrilakshimi et al^16^ incurred points deductions due to possible biases stemming from deviations in intended interventions and probable bias in outcome measurement. Amitha et al^17^ lost points related to bias in outcome measurement.

**Table 2:** Critical appraisal according to the RoB-2 tool for assessing the risk of bias in randomized controlled trials.

Study	Bias from randomization process	Bias due to deviations from intended interventions	Bias due to missing outcome data	Bias in measurement of the outcomes	Bias in selection of the reported result	Overall risk of bias
Gupta 2014	Low	Low	Low	Low	Low	Low
Kaur 2017	Low	Low	Low	Low	Low	Low
Akhila 2017	Low	Low	Low	Low	Low	Low
Amitha 2017	Low	Low	Low	Some concerns	Low	Some concerns
Deepa 2018	Some concerns	Some concerns	Low	High	Low	High
Gitte 2022	Low	Some concerns	Low	High	Low	High
Ishan 2022	Low	Low	Low	Low	Low	Low
Gowrilakshmi 2023	Low	Some concerns	Low	High	Low	High

**Figure 5:**
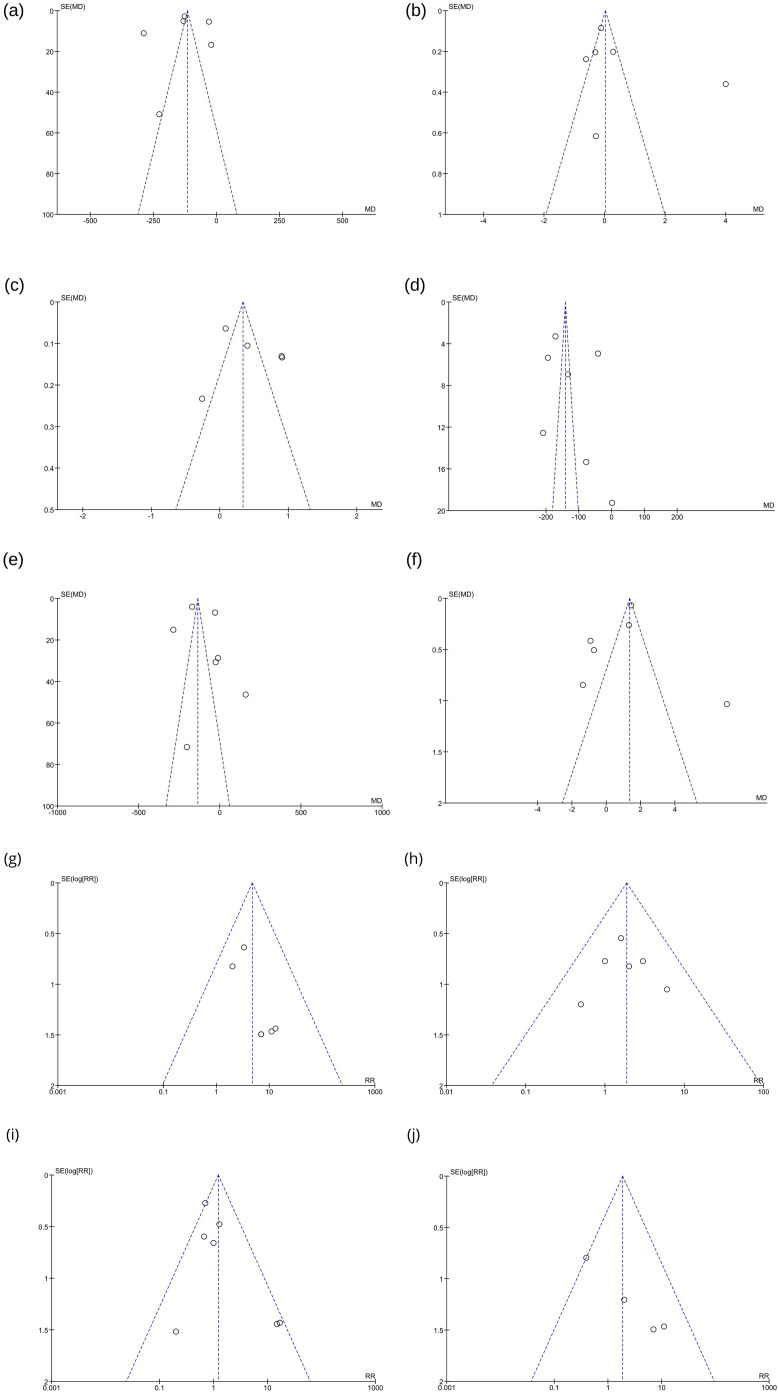
Publication bias assessment (funnel plots) of outcomes: (a) time to sensory regression to S1; (b) Onset time of motor block; (c) Onset time of sensory block; (d) Duration of motor block; (e) Duration of analgesia; (f) Time to achieve the highest sensory level; (g) Postoperative nausea and vomiting; (h) Bradycardia; (i) Hypotension; (j) Shivering.

Funnel plots for adverse effects displayed patterns consistent with low publication bias. However, an examination of time-related endpoints revealed a notable tendency toward bias. Comprehensive funnel plots for publication bias analysis are provided in Figure 5, in the Supplementary Material.

## Discussion

This systematic review and meta-analysis, involving 8 RCTs and 604 patients, compared buprenorphine and dexmedetomidine as adjuvants in spinal anesthesia for lower abdominal, pelvic, and lower limb surgeries. Key findings include: 1) Reduction in the time of sensory block regression to S1 in the buprenorphine group; 2) Shorter duration of motor block in the buprenorphine group; 3) Extended onset time of sensory block in the buprenorphine group; 4) Higher incidence of PONV when buprenorphine was used instead of dexmedetomidine as a neuraxial adjuvant. The deliberate inclusion of only RCTs, excluding observational studies, was aimed at enhancing the overall quality of the meta-analysis. Notably, this meta-analysis appears to be the first to attempt a direct comparison between buprenorphine and dexmedetomidine as adjuvants for spinal anesthesia.

Buprenorphine stands out due to its distinctive profile characterized by a high affinity for the three primary opioid receptors (µ, κ, and δ), coupled with a lower affinity for ORL-1 (Opioid-Receptor-Like-1).^18^^,^^19^ This unique binding pattern is instrumental in reducing the likelihood of tolerance and addiction when compared to full µ-opioid agonists. Particularly noteworthy is its prolonged binding to µ-opioid receptors and activation of ORL-1, which may contribute significantly to this advantageous effect.^20^ Moreover, recent research suggests that its extended duration of action is attributed to its newly discovered local anesthetic properties.^21^

In a meta-analysis of White et al,^22^ authors compared the efficacy and incidence of adverse effects of intravenous buprenorphine with morphine in acute pain management. They concluded that buprenorphine was an equally effective analgesic agent, capable of being an alternative opioid for this purpose. Although the authors only considered intravenous buprenorphine, the comparison of its analgesic potency with morphine provides a basis for understanding its power as an opioid in perioperative use, including intrathecal use. Still, there was an equal incidence of side effects in the buprenorphine group when compared with patients who received morphine.

Feenstra et al^23^ recently performed a meta-analysis comparing opioid-free with opioid-based anesthesia regarding Postoperative Nausea and Vomiting (PONV), concluding that PONV has occurred less in the first group. This adverse effect is directly linked to the rostral spread of opioids following intrathecal administration, which may lead to nausea, vomiting, and respiratory depression, as indicated by previous studies.^11^ This aligns with the outcomes of our meta-analysis, where the comparison of adverse effect incidence in the buprenorphine and dexmedetomidine groups revealed a higher prevalence of PONV in the buprenorphine group (RR = 4.06; 95% CI 1.80 to 9.18; *p* = 0.0008; I^2^ = 0%; Figure 3 a). Despite the previous study of Roberts et al^24^ that related a strong logarithmic dose-response relationship between postoperative opioid dose and PONV, when scrutinizing subgroups of the present study based on buprenorphine dosing, no discernible trend toward increasing dosing and subsequent rise in adverse effect incidence was evident.

When compared to placebo, intrathecal dexmedetomidine has been associated with prolonged duration of sensory block, greater duration of motor block, and increased time to first analgesic request.^25^ Another meta-analysis has found a relationship between increasing the dose of intrathecal dexmedetomidine and prolongation of the action of spinal anesthesia, with the risk of bradycardia increasing at the same time.^26^ However, it was not possible to identify in our meta-analysis a statistically different risk of bradycardia between groups with buprenorphine and dexmedetomidine.

Intrathecal dexmedetomidine, through its mechanism of action as an α2 receptor agonist in the dorsal horn of the spinal cord, proves valuable in extending neuraxial and peripheral nerve blocks. This quality positions it as an excellent adjuvant for enhanced analgesic efficacy.^27^ Furthermore, it demonstrates ability to prolong the duration of neuraxial blockade and improve postoperative analgesia without inducing significant adverse effects such as hypotension when administered at dosages up to 5 µg. Evidential support indicates a reduction in the need for postoperative analgesic rescue within the initial 24 hours, with 75% of patients not requiring additional analgesia in the dexmedetomidine group.^12^^,^^28^

Another clinical trial has provided support for the efficacy of intrathecal dexmedetomidine as an analgesic, sympatholytic, and sedative drug, all without inducing respiratory depression.^29^ In terms of analgesic potency, it has been shown to offer five times more potent analgesia than spinal fentanyl.^30^ Additionally, dexmedetomidine exhibits greater hemodynamic stability when compared to buprenorphine.^16^ Partially supported by the current systematic meta-analysis, earlier research findings from other studies have consistently shown prolonged analgesia times with dexmedetomidine^29^ and extended duration of sensory and motor block,^12^, ^16^ and a reduction in the onset time of both sensory and motor block.^29^

When subgroup analysis was performed to explore potential sources of heterogeneity in time-related outcomes, the primary endpoint “time to sensory regression to S1” did not reveal a clear dose-dependent effect of increasing local anesthetic doses, or a significant interaction with the use of adjuvants (buprenorphine or dexmedetomidine) or type of local anesthetic. We hypothesize that these findings, including high heterogeneity, may be influenced by methodological variation in the measurement of continuous variables and the relatively small sample sizes in some studies. Therefore, definitive conclusions regarding the impact of local anesthetic dose, adjuvants, or type of local anesthetic on time to sensory regression to S1 await further investigation in adequately powered randomized clinical trials.

The present study has certain limitations. There is a notable potential for publication bias, particularly in the analysis of time-related outcomes. Several factors may contribute to this bias, including selection bias, the file drawer effect, or reporting bias. Additionally, the limited number of RCTs for analysis can be attributed to the relatively short period since buprenorphine was first used as an off-label drug for spinal anesthesia. Furthermore, it is crucial to recognize that both buprenorphine and dexmedetomidine have not yet gained full acceptance from public agencies for use as neuraxial adjuvants in intrathecal anesthesia up to the present moment.^8^

## Conclusion

In conclusion, buprenorphine was deemed inferior to dexmedetomidine in maintaining sensory block, as evidenced by a reduced time to sensory regression to S1. Conversely, buprenorphine was associated with an increased incidence of PONV. Buprenorphine as a neuraxial anesthesia adjuvant may be a viable option when dexmedetomidine is unavailable or contraindicated. Additionally, ongoing research is essential for developing new drugs for spinal anesthesia, providing additional options for anesthesiologists, and bolstering evidence for the use of existing drugs. Further studies are warranted to determine the optimal doses of buprenorphine and dexmedetomidine for spinal anesthesia.

## Declaration of competing interest

The authors declare no conflicts of interest.

## Appendix Supplementary materials

Image, application 1

## Footnotes

Supplementary material associated with this article can be found, in the online version, at doi:10.1016/j.bjane.2024.844557.
