# Supplementary material for: The efficacy of buprenorphine compared with dexmedetomidine in spinal anesthesia: a systematic review and meta-analysis
Source: Braz J Anesthesiol. 2024 Sep 8;74(6):844557. doi: 10.1016/j.bjane.2024.844557 (PMC11465144; doi:10.1016/j.bjane.2024.844557)

**BJAN-D-24-00196_Supplementary Material**

**Figure Supplementary 5** Publication bias assessment (funnel plost) of time-related outcomes: (a) Time to sensory regression to S1; (b) Onset of motor block; (c) Onser of sensory block; (d) Duration of motor block; (e) Duration of analgesia; (f) Time to achieve the highest sensory level.

(a) (b)


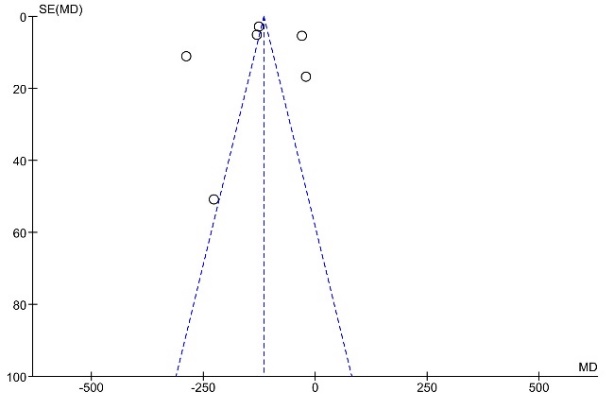

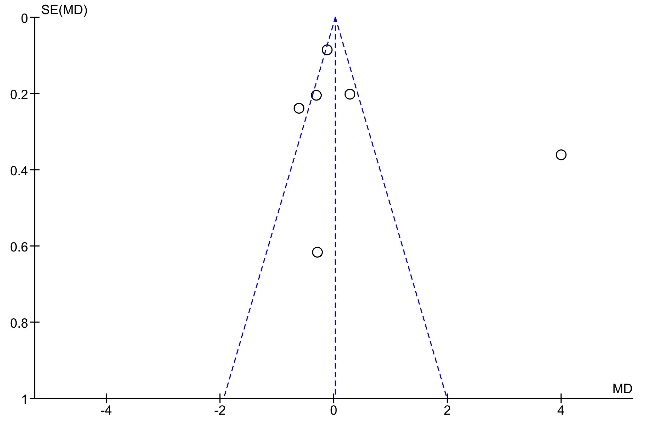


(c) (d)


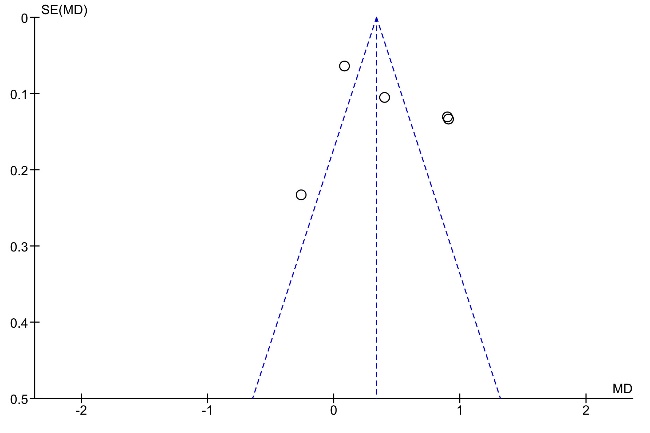

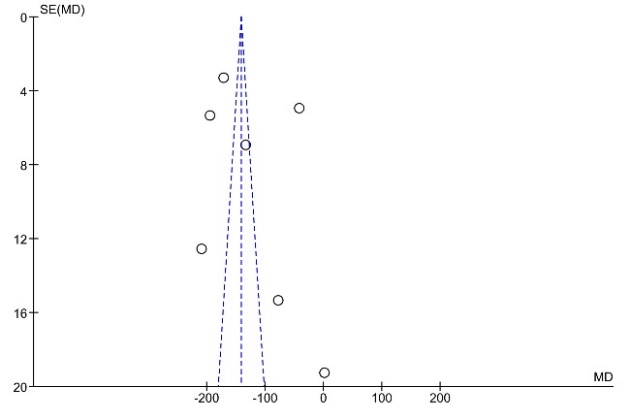


(e) (f)


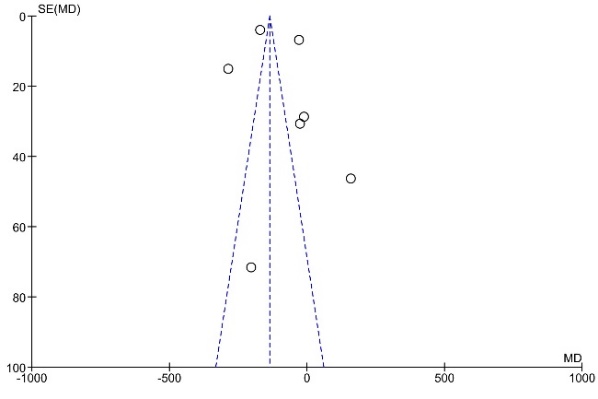

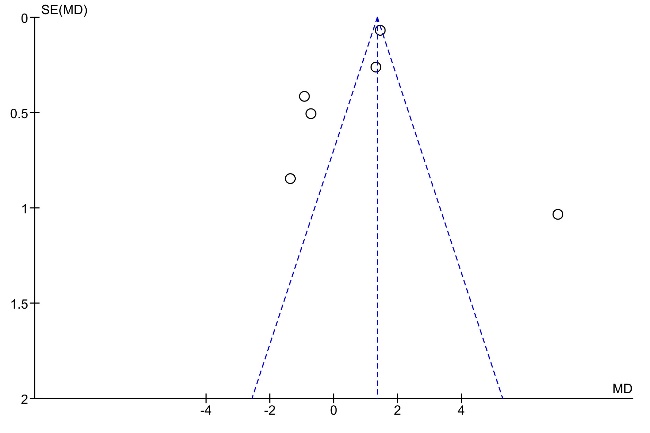

Supplement: Supplementary file 1 [file mmc1.docx]
